# Characteristic MicroRNAs Linked to Dysregulated Metabolic Pathways in Qatari Adult Subjects With Obesity and Metabolic Syndrome

**DOI:** 10.3389/fendo.2022.937089

**Published:** 2022-07-22

**Authors:** Fayaz Ahmad Mir, Raghvendra Mall, Ahmad Iskandarani, Ehsan Ullah, Tareq A. Samra, Farhan Cyprian, Aijaz Parray, Meis Alkasem, Ibrahem Abdalhakam, Faisal Farooq, Abdul-Badi Abou-Samra

**Affiliations:** ^1^ Qatar Metabolic Institute, Academic Health System, Hamad Medical Corporation, Doha, Qatar; ^2^ Qatar Computing Research Institute (QCRI), Hamad Bin Khalifa University, Doha, Qatar; ^3^ Department of Immunology, St. Jude Children’s Research Hospital, Memphis, TN, United States; ^4^ College of Medicine, Qatar University (QU) Health, Qatar University, Doha, Qatar; ^5^ Qatar Neuroscience Institute, Academic Health System, Hamad Medical Corporation, Doha, Qatar

**Keywords:** miRNA, metabolic disorder, HbA1c, obesity, mirDIP, network analysis

## Abstract

**Background:**

Obesity-associated dysglycemia is associated with metabolic disorders. MicroRNAs (miRNAs) are known regulators of metabolic homeostasis. We aimed to assess the relationship of circulating miRNAs with clinical features in obese Qatari individuals.

**Methods:**

We analyzed a dataset of 39 age-matched patients that includes 18 subjects with obesity only (OBO) and 21 subjects with obesity and metabolic syndrome (OBM). We measured 754 well-characterized human microRNAs (miRNAs) and identified differentially expressed miRNAs along with their significant associations with clinical markers in these patients.

**Results:**

A total of 64 miRNAs were differentially expressed between metabolically healthy obese (OBO) versus metabolically unhealthy obese (OBM) patients. Thirteen out of 64 miRNAs significantly correlated with at least one clinical trait of the metabolic syndrome. Six out of the thirteen demonstrated significant association with HbA1c levels; miR-331-3p, miR-452-3p, and miR-485-5p were over-expressed, whereas miR-153-3p, miR-182-5p, and miR-433-3p were under-expressed in the OBM patients with elevated HbA1c levels. We also identified, miR-106b-3p, miR-652-3p, and miR-93-5p that showed a significant association with creatinine; miR-130b-5p, miR-363-3p, and miR-636 were significantly associated with cholesterol, whereas miR-130a-3p was significantly associated with LDL. Additionally, miR-652-3p’s differential expression correlated significantly with HDL and creatinine.

**Conclusions:**

MicroRNAs associated with metabolic syndrome in obese subjects may have a pathophysiologic role and can serve as markers for obese individuals predisposed to various metabolic diseases like diabetes.

## Introduction

The worldwide rise in obesity and its strong association with metabolic diseases have elicited interest in the underlying mechanisms. According to the WHO report 2021, worldwide obesity has nearly tripled since 1975 (1). In 2016, more than 1.9 billion adults, 18 years and older, were overweight and over 650 million were obese (1). The global obesity epidemic is causing an alarming incidence of metabolic disorders. Obesity can be considered a growing epidemic that is associated with hyperglycemia (elevated blood glucose levels >7.0 mmol/L or hemoglobin that is glycosylated HbA1c > 6.5%), insulin resistance, and dyslipidemia (characterized by elevated cholesterol, low-density lipoproteins (LDL) and decreased serum high-density lipoproteins (HDL)), collectively referred to as metabolic syndrome (2). However, there are subjects with an elevated body mass index (BMI) who do not progress to metabolic syndrome; they are generally labeled as “Metabolically Healthy Obese” (2–7), they have obesity only (OBO); but the protective mechanisms are unknown. Body fat distribution is suspected to play an important role (8). High liver fat content and predominantly abdominal adiposity were shown to be linked to the metabolically unhealthy obesity phenotype (obesity with metabolic syndrome or OBM), whereas subcutaneous adiposity is associated with the metabolic healthy obesity phenotype (9, 10). Over the past years, some biological mechanisms and phenotypic characteristics have been identified that differentiate individuals with OBO from OBM (11). The concept of OBO may serve as a model to better understand the pathways and mechanisms linking obesity to metabolic diseases. Therefore, considering the potentially devastating impact of obesity, there is urgency in elucidating underlying mechanisms and identifying novel markers for risk stratification and targeted early treatment.

Impaired adipose tissue metabolism and function are central to the pathogenesis of obesity and associated metabolic disorders. MicroRNAs (miRNAs) play a crucial role in regulating gene expression and are likely to have an essential function in the pathogenesis of obesity and metabolic disorders (12). MicroRNAs are small non-coding RNAs participating in the post-transcriptional regulation of genes by negatively regulating them. Evidence is accumulating that circulating miRNAs, released by many types of cells act as a new class of endocrine factors. MiRNAs might serve as endocrine and paracrine messengers that facilitate communication between donor cells and tissues with receptor cells or target tissues, thereby potentially having important roles in metabolic organ crosstalk (13). In response to various pathophysiological conditions, miRNAs can be released by cells into their environment transported by different extracellular fluids, including blood, and could serve as biomarkers of diverse diseases including diabetes and related metabolic disorders. The role of miRNAs as key regulators of metabolic homeostasis has been intensely explored over the last decade. *Brando et al.* have collated the significant circulating miRNAs that are altered in obese subjects, where microRNAs such as miR-92a-3p, miR-122, miR-122-5p, miR-140-5p, miR-142-3p, miR-151a, miR-155, miR-222, and miR-15a have been shown to be upregulated. On the other hand miR-15a, miR-26a, miR-30b, miR-30c, miR-125b, miR-126, miR-139-5p, miR-144-5p, miR-146a, miR-150, miR-223, and miR-376a are reported to be downregulated in obese adults when compared to healthy lean individuals (14). Controversially, the role of miR-15b remains obscure where it has been upregulated in one study (15) while another group reported downregulation of miR-15b in obese subjects compared to lean counterparts (16). Although obesity is linked to differentially expressed miRNAs, they additionally contribute to various metabolic disorders including hypertension, hepatic steatosis, and insulin resistance by influencing the metabolism of cholesterol, LDL (mir-26a and mir-15b), and elevation of circulating glucose (mir-140-5p, miR-142-3p, miR-222, and mir125b) that eventually glycosylates hemoglobin (HbA1c) respectively (12, 14, 17–21).

**Figure 1:**
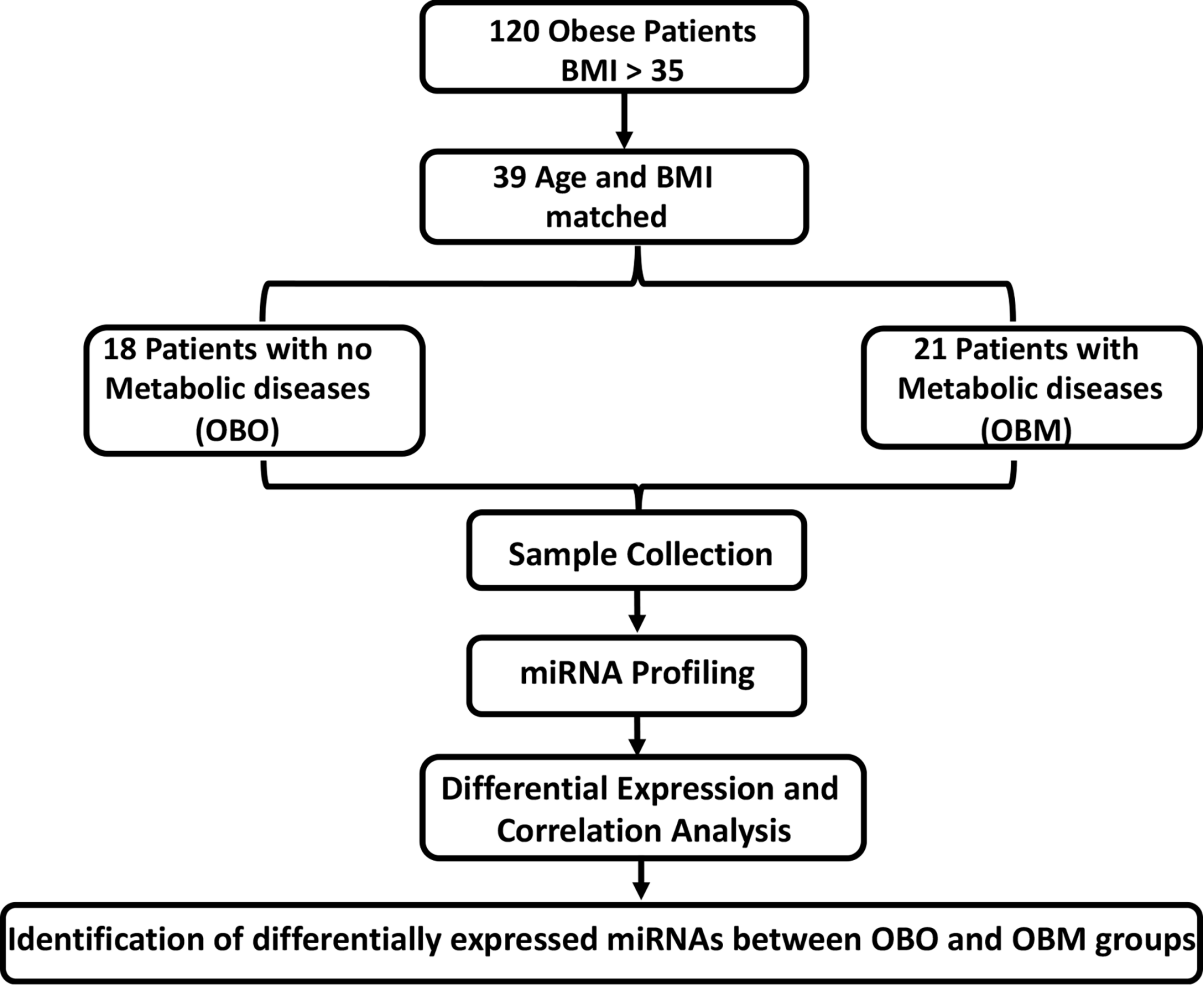
The experimental study design. BMI, Body Mass Index; OBO, Obesity with no metabolic disease; OBM, Obesity with metabolic diseases. To determine the fold-changes of miRNA expression between the OBO vs OBM patients, we used the Relative Quantification (RQ) measure. We considered those miRNAs to be differentially expressed for which |log2(RQ)| > 2 and significance threshold p< 0.05. This resulted in the identification of 64 differentially expressed miRNAs. Out of the 64 miRNAs, there were 13 miRNAs whose expression correlated with at least one clinical trait of relevance for metabolic syndrome (including HBA1c, Creatinine, Cholesterol, LDL, and HDL).

Here, we aimed to unravel the associations between the metabolic parameters in obese individuals with miRNA profiling. We identified 64 significantly differentially expressed miRNAs of which 36 were down-regulated and 28 were up-regulated. By undertaking an association discovery approach, we identified the expression of eleven out of the 36 down-regulated miRNAs and two of the 28 up-regulated miRNAs in our patient dataset were significantly correlated with at least one clinical trait of relevance to metabolic syndrome. The down regulated miRNAs include miR-106b-3p, miR-103a-3p, miR-130b-5p, miR-153-3p, miR-182-5p, miR-331-3p, miR-363-3p, miR-433-3p, miR-636, miR-652-3p and miR-93-5p whereas miR-452-3p and miR-485-5p were upregulated. Several of the miRNAs in OBM patients were significantly dysregulated and associated with increased levels of HbA1c and cholesterol. These include miR-130b-5p, miR-153-3p, miR-182-5p, miR-331-3p, miR-363-3p, miR-433-3p, miR-452-3p, miR-485-5p and miR-636. **Figure 1** provides an outline of our experimental design.

## Materials and methods

### Study Design

The participants were recruited at the Qatar Metabolic Institute, Hamad Medical Corporation, Doha, Qatar. The study protocol was approved by the institutional review board (IRB) of Hamad Medical Corporation (HMC, IRB protocol #16245/16) and all participants provided written informed consent. Obesity was determined according to CDC guidelines. Both Class 1 (BMI of 35 to 40) and Class 2 (BMI > NA). Both Class 1 and Class 2 obesity were referred to as morbid obesity. A total of 120, male and female participants aged between 18 to 65 years with morbid obesity (BMI≥35 kg/m^2^) were included. Individuals such as pregnant females and those with identified chronic disease or terminal illness were excluded from the study. The subjects were classified into two groups those without metabolic syndrome (OBO) and with metabolic syndrome (OBM) components of the metabolic syndrome; obesity PLUS any 2 of the following: triglycerides ≥ 150 mg/dL (1.7 mmol/L), HDL< 40 mg/dL (1.03 mmol/L) in men or< 50 mg/dL (1.29 mmol/L) in women, blood pressure ≥ 130/85 mmHg and fasting blood glucose ≥ 110 mg/dL (5.6 mmol/L) (22). An additional, filter of age and BMI matching yielded 39 subjects that consisted of 18 OBO subjects and 21 OBM subjects. Among the 18 OBO group subjects, none of the subjects had hyperglycemia, 2 individuals were identified with hypertension, 2 with mildly elevated triglycerides, and 6 with a borderline decrease in HDL. Venous blood samples were collected from these 39 subjects for total miRNA isolation.

### Participants Characteristics

Height and weight were measured in light clothing without shoes. Fasting blood samples were taken between 7-9 AM after at least 12h of fasting. For serum collection, whole blood was collected *via* BD Vacutainer Serum Separation Tubes (BD Biosciences, Franklin Lakes, NJ, USA). Blood samples were kept at room temperature for 30-60 minutes and then centrifuges at 3000g for 10 minutes. Following centrifugation, serum was separated and immediately stored at -80°C for further use.

Blood biochemistry was performed at the HMC clinical laboratory which has been accredited by the College of American Pathologists (CAP). Measurements included HbA1c with Turbidimetric Inhibition Immunoassay (TINIA Roche Diagnostics, Mannheim, Germany), glucose by enzymatic reference method with hexokinase (Cobas 6000, Roche Diagnostics International, Switzerland), Total cholesterol, triglycerides, and high-density lipoprotein (HDL) cholesterol levels were measured enzymatically using a Synchron LX20 analyzer (Beckman-Coulter, High Wycombe, UK).

### RNA Isolation and Quality Control

Whole blood (2.5 ml) was collected into PaXgene Blood RNA Tubes (PreAnalytix). The tubes were inverted 8-10 times then placed at room temperature for at least 2 hours, frozen at -80°C, thawed overnight, then total RNA was isolated with a PAXgene Blood RNA Kit including the DNase Set (Qiagen). The concentrations and purity of the RNA samples were evaluated spectrophotometrically (Nanodrop ND-1000, Thermo, Wilmington, DE USA). The RNA isolation process was validated by analyzing the integrity of several RNAs with the RNA 6000 Nano Chip Kit (Agilent). The presence of the small RNA fraction was confirmed by the Agilent Small RNA Kit (Agilent).

### MicroRNA (miRNA) Profiling

The expression levels of 754 miRNAs were profiled using the TaqMan OpenArray Human MicroRNA panels (PN: 4470189; Life Technologies Forster City, CA, USA) on a QuantStudio 12K Flex instrument. For all experimental groups, 3 µL (~10 ng) of total RNA was used for reverse transcription (RT) reactions using MegaPlex RT Primers Human Pool Set v3.0 (PN: 4444745; Pool A v2.1 and Pool B v3.0) according to the manufacturer’s optimized protocol for low sample input for profiling human microRNA using the OpenArray platform on BioRad c1000 Touch thermal cycler. No-template controls were included. Pre-amplification of RT products was performed using a 5 µL RT reaction combined with the matching Megaplex PreAmp Primer Pool A v2.1 or B v3.0 and amplified using the thermal cycler (Applied biosystems). The pre-amplified products were diluted at 1:40 in 0.1x TE pH 8. For each experimental set, 10 µL of the diluted products were combined to give a total of 40 µL pooled sample. For both Pool A and Pool B groups, 22.5 µL of the pooled products were combined with an equivalent volume of TaqMan OpenArray Real-Time Master Mix and aliquoted into a 96-well plate. Then, 5 µL from each well were then transferred into a 384 well plate for loading onto OpenArray plates using an AccuFill robotic system. The OpenArray plates were run on a QuantStudio 12K Flex instrument (Life Technologies) and the raw data files were imported and analyzed using the DataAssist software (Life Technologies). Failed reactions were excluded from analysis and undetermined CT values for samples sets determined to have good amplifications were assigned a threshold value of 40, defining low abundance or absence of miRNA expression. Global mean normalization was used to calculate relative fold change for the miRNA expression.

### Statistics

Statistical characteristics of clinical measurements were calculated by comparing the OBO and OBM samples using R v4.2.0 (23). The normality of the measurements was tested using Anderson-Darling test using nortest v1.0.4 package (24). The *Student’s t-test* was used to calculate the p-value of the normally distributed measurements. For the remaining measurements, Mann-Whitney test from the base package in R was used. P-values were not corrected for false discovery rate (FDR) owing to the small sample size.

**Figure 2:**
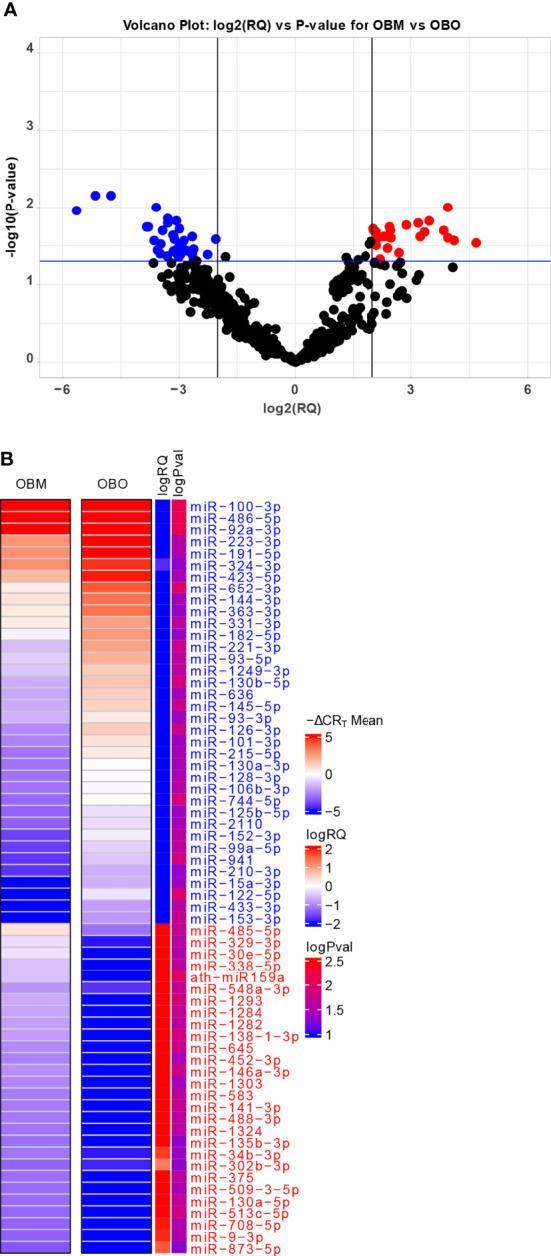
**(A)** Volcano Plot highlighting the differentially expressed microRNAs. The red-colored microRNAs are over-expressed in OBM versus OBO while the blue-colored microRNAs are under-expressed. Here ‘RQ’ is equivalent to the fold-change of a particular miRNA (Wang, Wang, and Xi 2011) and is ∝ mean -ΔCR_T_ values. **(B)** The mean -ΔCR_T_ values for the differentially expressed miRNAs for the OBM and OBO groups respectively. The -ΔCR_T_ values are ∝ to miRNA expression, where the higher -ΔCR_T_ value (or CR_T_ value) corresponds to higher miRNA expression levels. This is further reflected in the logRQ values which are equivalent to fold-change in the expression of individual miRNA. Here ‘logPval’ corresponds to -log10 (P-value).

The miRNA expression levels were measured *via* raw CR_T_ values, which are inversely proportional to miRNA expression i.e., the higher the CR_T_ value lower the expression of the circulating miRNA (25). However, current miRNA microarray platforms might not have enough miRNAs which are stably expressed as indicated in (26). Thus, to measure fold-changes in miRNA expression, we determined the Relative Quantification (RQ) values using the standard formula (27). An RQ value showcased the fold-change (FC) of a specific miRNA in two populations. An RQ=1 indicated that a specific miRNA was not differentially expressed in the OBO versus OBM samples. Otherwise, if the |log2(RQ)| > 2 and significance threshold (p< 0.05), then the miRNA was differentially expressed between the two groups as observed in **Figure 2A**.

### Visualizations

The volcano and scatter plots were constructed using the ggplot2 v3.3.6 package in R. The visualization of the miRNA expression matrix was performed using the ComplexHeatmap v2.12.0 package (28) in R.

### Correlation Analysis

We performed a set of correlation analyses, where we correlated the expression (-CR_T_ value when available) of each differentially expressed miRNAs with the different clinical traits of relevance to metabolic syndrome including HBA1c, Creatinine, Cholesterol, LDL, and HDL. The correlations were estimated using the ‘cor.test’ function from the stats package using the Pearson correlation method. The correlations between differentially expressed miRNAs and clinical traits were visualized using the corrplot v0.92 package (https://github.com/taiyun/corrplot).

Additionally, we visualize the significantly correlated miRNAs’ expression versus individual clinical trait values for the OBO and OBM patients through a scatter plot. We fit a linear regression line along with confidence intervals using the ‘geom_smooth’ function and annotate the Pearson correlation scores and p-values in the plot using the ‘stat_cor’ function from the ggplot2 package.

### MiRNA-mRNA Interaction Network

We used the microRNA Data Integration Portal, mirDIP v4.1 (http://ophid.utoronto.ca/mirDIP/), which provides nearly 152 million human microRNA–target predictions collected from 30 different resources (21). The mirDIP integrative score was constructed by taking a statistical consensus from the predictions available through myriad resources and was assigned to each unique miRNA-target interaction to provide a unified measure of confidence. The integrated scores range, 0 to 1, was used; higher scores correspond to stronger evidence of potential interaction between miRNA and target gene; the target genes were thus identified.

### Pathway Enrichment Analysis

The mRNAs which were identified to be regulated by the differentially expressed miRNAs were then utilized in an overexpression analysis framework. We used the ConsensusPathDB (29) web portal (http://cpdb.molgen.mpg.de/) as utilized in (30–34) to identify significantly enriched pathways choosing the PID (http://pid.nci.nih.gov/) and KEGG (https://www.genome.jp/kegg/pathway.html) database. We also used the ConsensusPathDB web-portal to determine the significantly enriched GO terms. The significantly enriched pathways and GO terms were determined using a hypergeometric test.

The hypergeometric test was performed as described below. Let the total number of genes associated with our differentially expressed miRNAs be *n*. Out of these *n*, say *k* genes are part of a pathway (p). This pathway (p) consists of a total of *K* genes. The total number of background genes (or all protein-coding genes in humans) be *N*. Then, the probability of significance of the pathway can be determined by the hypergeometric test as follows:


$$P\left(p\right)=\;\frac{\left(\begin{array}{c} K\\ n \end{array}\right)\left(\begin{array}{c} N-K\\ n-k \end{array}\right)}{\left(\begin{array}{c} N\\ n \end{array}\right)}$$

where $\left(\begin{array}{c} N\\ n \end{array}\right)$ represents the combination function (35).

## Results

### Clinical Characteristics

The clinical characteristics of the study subjects are summarized in **Table 1**. The clinical traits that were significantly different between the two groups include HbA1c (p=0.002), triglycerides (p=0.001), high-density lipoprotein (HDL, p=0.008), glucose (p=0.009), and insulin (p=0.05). Other important clinical traits which were not significantly different between the two sets include clinical variables such as creatinine, low-density lipoprotein (LDL), and cholesterol.

**Table 1:** Clinical and biochemical traits of the study subjects.

Feature	OBO	OBM	P Value
Age (years)	38.06 ± 4.21	40.52 ± 7.26	0.283
Females (N)	11	9	
Males (N)	7	12	
Height (cm)	167.4 ± 11.9	170.8 ± 9.6	0.370
Weight (kg)	113.4 ± 19.6	110.9 ± 27.6	0.782
BMI (kg/m^2^)	40.0 ± 4.5	39.6 ± 3.0	0.746
Smoking (%)	6.0	33.0	
HbA1c (%)	5.5 ± 0.27	7.02 ± 1.9	0.002
TG (mmol/L)	1.39 ± 0.48	2.65 ± 1.52	0.001
Cholesterol (mmol/L)	4.9 ± 1.1	4.8 ± 1.1	0.855
LDL (mmol/L)	2.8 ± 1.3	2.6 ± 1.1	0.728
HDL (mmol/L)	1.5 ± 0.7	1.0 ± 0.3	0.008
Glucose (mmol/L)	5.2 ± 0.6	7.4 ± 3.4	0.009
Creatinine (mmol/L)	67.5 ± 14.1	65.3 ± 14.1	0.563
Insulin (miU/mL)	19.0 ± 13.3	27.6 ± 13.2	0.053
CRP (mg/L)	12.8 ± 12.5	7.1 ± 4.5	0.064
ALT (U/L)	20.7 ± 11.6	36.5 ± 35.1	0.063
AST (U/L)	18.8 ± 9.6	23.6 ± 15.0	0.251

OBO (obesity only), and OBM (obesity with metabolic syndrome). Significance was determined by the Student’s t-test.

### Differential Expression Analysis

We identified a total of 64 miRNAs to be differentially expressed between the OBO and OBM groups (**Figures 2A, B** and **Supplement Table 1**) of which 36 miRNAs were down-regulated and 28 were up-regulated in the OBM patients when compared to the metabolically healthy obese (OBO) patients (**Figure 2A**). Specific miRNAs; miR-873-5p (-ΔCR_T_ = 1.62), miR-9-3p (-ΔCR_T_ = 1.91), mir-708-5p (-ΔCR_T_ = 1.96) were significantly up-regulated in OBM (had higher mean -ΔCR_T_) in comparison to OBO patients (**Figure 2B** and **Supplementary Table 1**). On the contrary, miRNAs; miR-100-3p (-ΔCR_T_ = -9.18), miR-486-5p (-ΔCR_T_ = -5.16) and miR-92a-3p (-ΔCR_T_ = -4.76), were among the most significantly downregulated miRNAs in OBM versus OBO patients.

### Correlations With Metabolic Syndrome Relevant Clinical Markers

**Figure 3:**
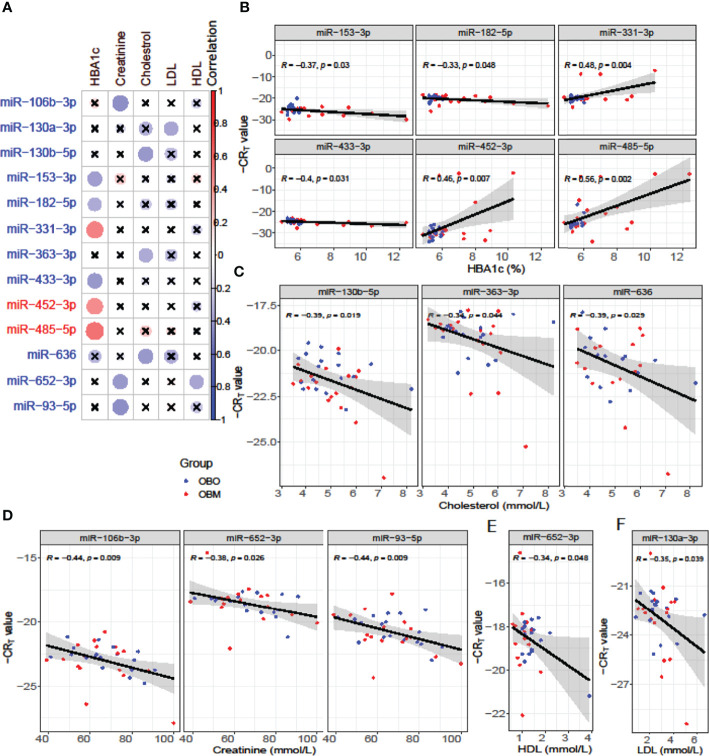
**(A)** Pearson correlation between clinical traits relevant to metabolic syndrome and miRNA expression (-CR_T_ values). The ‘x’ represents that the correlation coefficient is not significant. The darker the correlation coefficient (‘red’ or ‘blue’) the stronger the correlation (more towards +1 or more towards -1). Significant correlations (p< 0.05) of clinical traits with relevance to metabolic syndrome with the differentially expressed miRNAs. **(B)** Correlation with HBA1c; **(C)** Correlation with Cholesterol; **(D)** Correlation with Creatinine; **(E)** Correlation with HDL, and **(F)** Correlation with LDL.

We next performed a set of correlation analyses, where we correlated the expression (-CR_T_ value when the measurement was available) of each differentially expressed miRNAs with clinical lab traits of relevance to metabolic syndrome including HbA1c, creatinine, cholesterol, LDL, and HDL. 11 out of 36 down-regulated miRNAs and 2 out of 28 up-regulated miRNAs correlated significantly (p< 0.05) with at least one of the clinical lab traits (**Figure 3A**). The correlation values across these 13 miRNAs and 5 clinical traits are summarized in **Table 2**. As depicted in **Figure 3A** the miRNAs miR-153-3p, miR-182-5p, and miR-433-3p correlated negatively, while miR-331-3p, miR-452-3p, and miR-485-5p demonstrated a positive correlation with HbA1c. Interestingly, the trend for miRNAs: miR-153-3p, miR-182-5p, and miR-433-3p, the -CR_T_ values decreased linearly with higher (dysregulated) levels of HbA1c (**Figure 3B**). This trend was distinct for the OBM patients, suggesting the loss of expression of these miRNAs in OBM patients was significantly related to increased (↑) HbA1c levels. Similarly, from **Figure 3B** for HbA1c, we could also observe another trend for miRNAs: miR-331-3p, miR-452-3p, and miR-485-5p. The -CR_T_ values of these miRNAs went significantly up i.e., these miRNAs were significantly over-expressed in OBM patients with increased HbA1c levels.

**Table 2:** Pearson correlation coefficients of the clinical traits associated with metabolic syndrome with the miRNA expression of relevant differentially expressed miRNAs.

Diff MiRNAs	HBA1c	Creatinine	CHOLESTROL	HDL	LDL
miR-106b-3p	0.161	**-0.436**	-0.0776	-0.182	0.001
miR-130a-3p	-0.0913	-0.161	-0.309	0.0669	**-0.351**
miR-130b-5p	-0.111	-0.026	**-0.388**	-0.0154	-0.28
miR-153-3p	**-0.372**	0.219	-0.115	0.168	-0.152
miR-182-5p	**-0.327**	-0.0811	-0.225	0.0626	-0.229
miR-331-3p	**0.484**	-0.0371	0.0247	-0.197	0.0653
miR-363-3p	0.0575	-0.0793	**-0.338**	-0.064	-0.268
miR-433-3p	**-0.395**	0.0775	-0.13	-0.0304	-0.113
miR-452-3p	**0.456**	-0.059	0.0267	-0.183	0.0224
miR-485-5p	**0.555**	0.0405	0.221	-0.0822	0.152
miR-636	-0.282	-0.103	**-0.393**	-0.0614	-0.337
miR-652-3p	0.0627	**-0.383**	-0.00376	**-0.342**	0.0976
miR-93-5p	-0.0617	**-0.437**	-0.033	-0.231	0.0828

The bold values represent strong correlations i.e. |correlation| > 0.3.

We further identified that miRNAs: miR-106-3p, miR-652-3p, and miR-93-5p were significantly correlated with creatinine levels of patients in our dataset, and miRNAs: miR-130b-5p, miR-363-3p, and miR-636 were significantly associated with the cholesterol levels of patients as observed in **Figure 3**. However, the ability to distinguish OBM patients from OBO patients through the -CR_T_ values of these miRNAs was not as stark as of those miRNAs associated with HBA1C (see **Figures 3D, C** respectively). This can also be attributed to the fact that creatinine and cholesterol levels were not significantly different between the two groups as indicated in **Table 1**. We also identified a miRNA, mir-652-3p, that was significantly negatively correlated with LDL (R = -0.34, see **Figure 3E**). Interestingly, the majority of OBM patients had lower LDL values as well as higher expression of mir-652-3p, and the majority of OBO patients had higher LDL values with lower expression of this miRNA. Lastly, we observe a significant negative correlation between mir-130a-3p expression and the clinical trait HDL (R = -0.35, see **Figure 3F**) with no clear distinction between the OBM and OBO groups.

**Figure 4:**
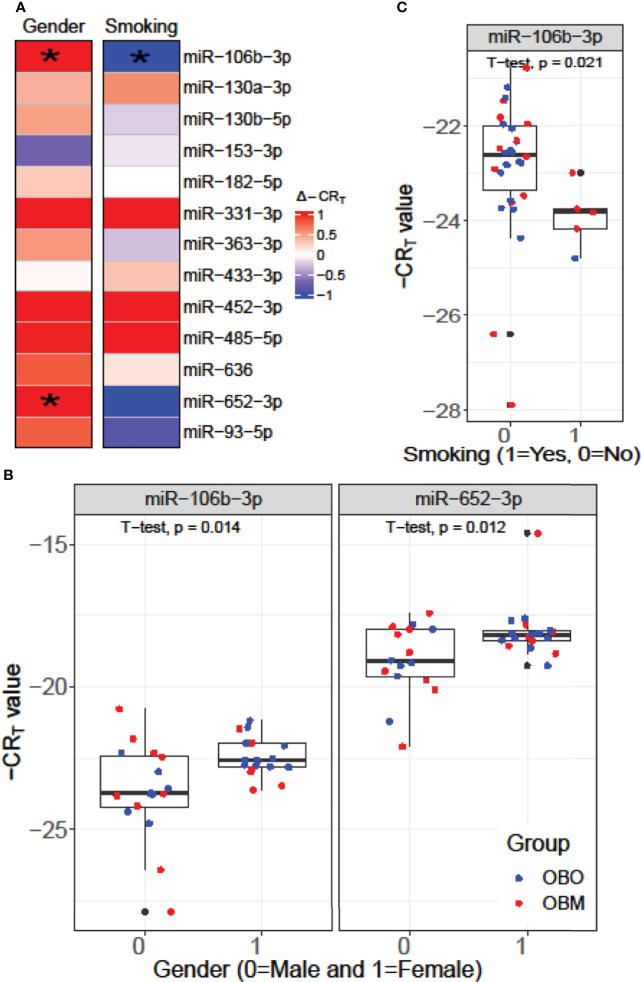
**(A)** Comparison of the expression pattern of the 13 differentially expressed miRNAs for Gender and Smoking status of patients using a *Student’s* t-test. Here ‘*’ represents a significant association (p< 0.05). **(B)** Boxplot illustrating the significant difference in expression of miR-106b-3p and miR-652-3p between males and females. **(C)** Boxplot highlighting the significant difference in expression of miR-106-3p between patients who smoke versus those who don’t.

We performed a *Student’s* t-test to determine whether the expression values of the 13 miRNAs of interest were significantly different between the males (Gender = 0) and females (Gender = 1) or smokers (Smoking 1 = Yes) versus non-smokers (Smoking 0 = No) in our dataset as illustrated in **Figure 4**. From **Figure 4A**, we observed that miR-106b-3p and miR-652-3p had significantly different expressions in males versus females, where both these miRNAs had lower expression in males when compared to females. Hence the difference in the -CR_T_ values are positive (Δ-CR_T_ > 0) as indicated in **Figure 4A**. However, for each of these miRNAs, there is no clear segregation of the expression of the miRNA between the OBO versus OBM male patients (Gender = 0) or female patients (Gender = 1) as observed in **Figure 4B**. This suggests that gender does not really have an impact on the differential expression of these miRNAs (miR-106b-3p and miR-652-3p) between the OBO and OBM patient groups.

From **Figure 4C**, we observe that miR-106b-3p has higher expression in patients who don’t smoke (0 = No) when compared to patients who smoke (1 = Yes) and the majority of the smokers (4 out of 6) belong to the OBM category. While miR-106b-3p is differentially expressed w.r.t. smoking status, there is no clear segregation of its expression between OBO and OBM groups, for patients who don’t smoke. Moreover, owing to the small sample size of patients with a positive smoking status (6 patients only), it is imperative not to draw strong conclusions. However, for a larger population size smoking would be a covariate to regress out when determining differentially expressed miRNAs for the phenotype of interest (i.e. OBO vs OBM patients).

### Mechanistic Insights from miRNA-mRNA Networks

**Figure 5:**
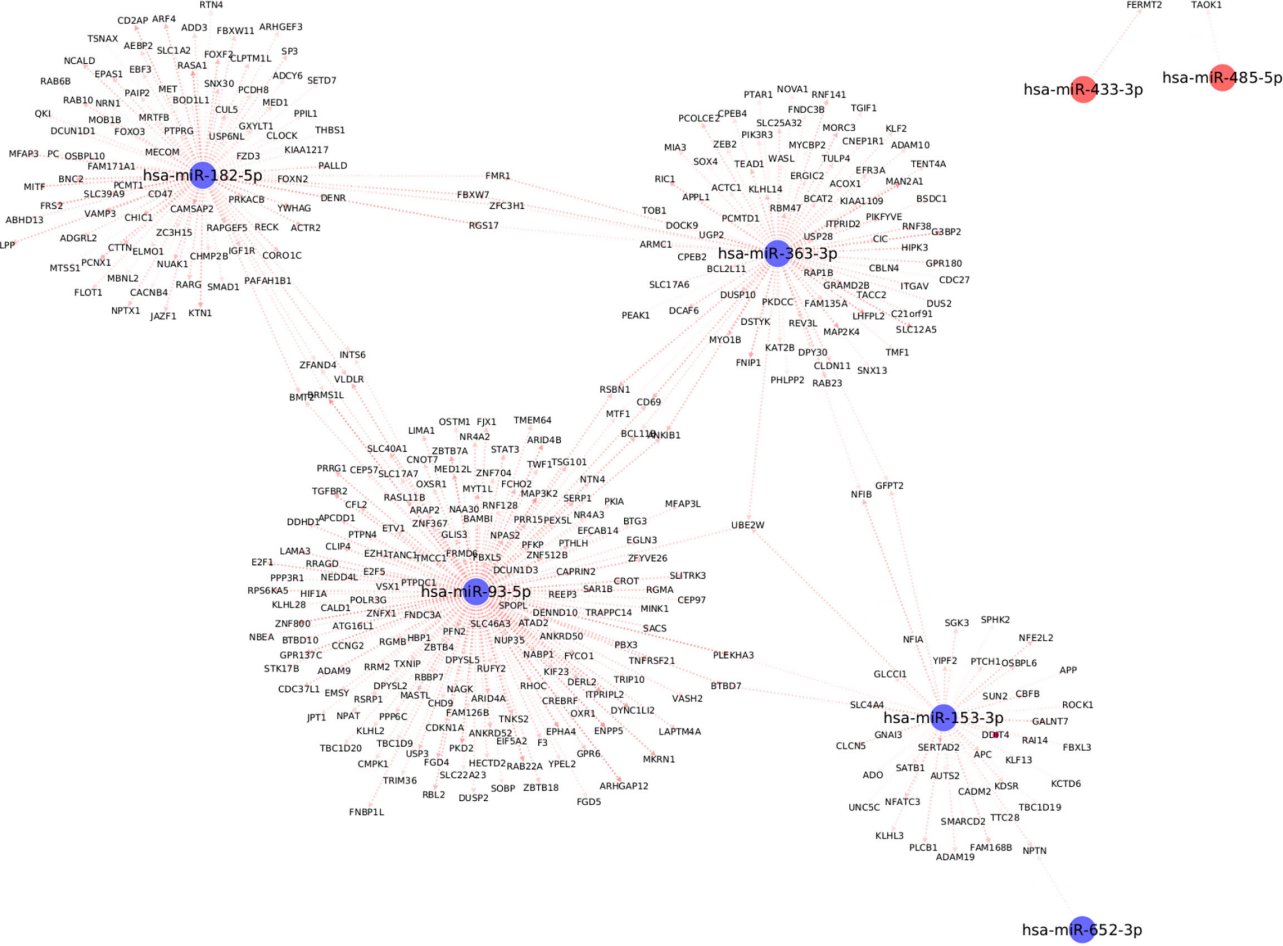
Top differentially expressed miRNAs with strong known interaction (coming from >=10 resources and interaction score>=0.75 from mirDIP) with target genes. Here we highlight only those miRNAs which are significantly correlated with at least one clinical trait relevant to metabolic syndrome.

We used the mirDIP database to extract information about target mRNAs which can be regulated by the differentially expressed miRNAs with significant associations with clinical traits. We use stringent cutoffs including a minimum of 10 resources and an integrated score of at least 0.75 to retain a potential interaction between miRNA and the target gene. This resulted in a total of 398 interactions between the seven (out of the 13) differentially expressed miRNAs and 378 target genes. Interestingly, we observed from **Figure 5**, that each of the seven differentially expressed miRNAs forms its own cluster of target genes with small overlaps amidst their interactomes. We then performed downstream pathway enrichment using overexpression analysis through ConsensusPathDB to identify significantly enriched pathways associated with each of these miRNAs. We could determine the enriched pathways and GO terms for four of these seven miRNAs. The top five significantly enriched pathways and top three biological processes, cellular components, and molecular functions for each of these miRNAs were detailed in **Supplementary Tables 2** and **3** respectively.

For example, the miRNA, miR-153-3p, is differentially downregulated with a ΔCʀтт of -3.4 (p=0.02, **Supplementary Table 1**); the target genes for this miRNA are SPHK2, GNAI3, ROCK1, and PLCB1; which are essential for the Sphingolipid signaling pathway (**Supplementary Table 2**). The Sphingolipid signaling pathway has been shown to play an important role in the regulation of obesity and type 2 diabetes (36). Similarly, the PDGFR-beta signaling pathway was significantly enriched based on the target genes of both miR-182-5p (USP6NL; CTTN; RASA1; ACTR2; YWHAG) and miR-363-3p (MAP2K4; WASL; ITGAV; RAP1B) respectively. The PDGFR-beta signaling pathway is known to play a role in the regulation of adipose progenitor maintenance and adipocyte-myofibroblast transitions (37). We identified BMP receptor signaling as one of the enriched pathways for miR-93-5p target genes (BAMBI; RGMB; RGMA). It has previously (38) been demonstrated that BMP signaling was relevant for both the white and brown adipogenesis and plays an important role when interconnecting obesity with metabolic and cardiovascular diseases. Finally, we identified the cellular senescence pathway from KEGG as a significantly enriched pathway for miR-93-5p target genes (TGFBR2; E2F5; E2F1; PPP3R1; CDKN1A; RBL2). Interestingly, recently *Smith, Ulf et al.* (39) reviewed that white adipose tissue cells are highly susceptible to becoming senescent both with aging, obesity and type 2 diabetes, independent of the chronological age. The white adipose tissue senescence is associated with the inappropriate expansion of adipocytes, insulin resistance, and dyslipidemia i.e., metabolic syndrome, a finding in line with our phenotype.

Additionally, we identified several different significantly enriched GO terms based on the target genes for each of the top four miRNAs (see **Supplementary Table 3**). These include GO terms associated with biological processes such as positive regulation of metabolic process (GO:0009893), cellular developmental process (GO:0048869), cellular protein modification process (GO:0006464), mitotic cell cycle process (GO:1903047) for miR-153-3p, miR-182-5p, miR-363-3p and miR-93-5p respectively (**Supplementary Table 3**).

## Discussion

Individuals with a persistently high BMI are at the risk of developing metabolic syndrome, a medical condition characterized by obesity, insulin resistance, dyslipidemia, and hypertension, with an accompanying risk of type 2 diabetes mellitus and cardiovascular disease (22). There is abundant literature that has investigated the metabolic differences underpinning lean and obese subjects (14). Obese individuals have been the focus of health care in recent years since the reversal of obesity by lifestyle, medical or surgical intervention protects them from metabolic syndrome (40). However, clinical observations identify a proportion of individuals with elevated BMI who led an active and healthy life relatively free of metabolic complications. This population is of particular interest and intensely investigated to elucidate the underpinning mechanisms and gene regulation that confer protection against the development of metabolic syndrome. Moreover, differentiation between OBO and OBM as well as early detection is paramount for clinical management of these individuals.

Several studies have identified the essential role of differentially expressed miRNA in obesity where a cluster of miRNAs; miR-92a-3p, miR-122, miR-122-5p, miR-140-5p, miR-142-3p, miR-151a, miR-155, miR-222, and miR-15a are upregulated and a group of miRNA; miR-15a, miR-26a, miR-30b, miR-30c, miR-125b, miR-126, miR-139-5p, miR-144-5p, miR-146a, miR-150, miR-223 and miR-376a are downregulated in obese adults (14). In this study, we focus particularly on morbidly obese individuals with a BMI>35kg/m^2^ who are metabolically protected and susceptible. Our results demonstrate that miR-106b-3p, miR-130a-3p, miR-130b-5p, miR-153-3p, miR-182-5p, miR-331-3p, miR-363-3p, miR-433-3p, miR-636, miR-652-3p, and miR-93-5p were significantly downregulated whereas miR-452-3p, and miR-485-5p were significantly upregulated in morbidly obese patients with metabolic diseases compared to obese patients without any metabolic disease in a dataset of Qatari population (**Figures 2** and **3**). These miRNAs significantly correlated with at least one clinical trait of relevance to metabolic syndrome like increased levels of HbA1c, creatinine, cholesterol, LDL, and HDL in our dataset (**Figure 3A**). The differentially expressed miRNAs correlate significantly with HbA1c (downregulated miR-153-3p, miR-182-5p and miR-433-3p; upregulated miR-331-3p, miR-452-3p and miR-485-5p), creatinine (downregulated miR-106b-3p, miR-652-3p and miR-93-5p), cholesterol (downregulated miR-130b-5p, miR-363-3p and miR-636), LDL (downregulated miR-130a-3p), and HDL (downregulated miR-652-3p) in our dataset in the context of OBO and OBM. Interestingly, we identify differential expression of miR-92a-3p, miR-122-5p, miR-15a, miR-125b, and miR-146a in our data which have previously been reported to be relevant for obesity (**Figure 2B** and **Supplementary Table 1**) (14).

Our unique dataset with the morbidly obese individuals (OBO and OBM) highlights various differentially expressed miRNAs which have been previously reported in obesity (14) conferring confidence in our study. Although identified in prior studies, these miRNAs (miR-92a-3p, miR-122-5p, miR-15a, miR-125b, and miR-146a) do not associate significantly with clinical lab traits, nor are they upregulated or downregulated in alignment with previous studies. This can be a result of our focus on metabolically healthy and unhealthy obese subjects compared to prior investigations that analyze lean and obese groups. Our findings further demonstrate that miR-153-3p, miR-182-5p, and miR-433-3p are downregulated in the OBM group and negatively correlated with HbA1c. Among these miRNAs, miR-153-3p has been reported to be overexpressed in lupus nephritis patients (28). Through miRNA-mRNA network analysis, we have shown that miR-153-3p regulates sphingolipid signaling. The sphingolipid pathway is known to be extensively involved in obesity and obesity-induced hyperglycemia (36). In agreement with our results, miR-182-5p has been reported to be suppressed in diabetes patients. Interestingly, it was shown that the expression of miR-182-5p is high in newly diagnosed patients compared to healthy control. However, its expression decreased with the increasing duration of T2DM (41). Another miRNA downregulated in the OBM group and positively correlated with HbA1c is miR-433-3p, which has been reported to be overexpressed in serum of hepatocellular carcinoma patients (42), pediatric beta-thalassemia patients, and needs further evaluation in the context of changes to hemoglobin (43). Moreover, miR-331-3p, which was downregulated in metabolically unhealthy obese patients and positively correlated with HbA1c has been reported as a biomarker for HCV-related hepatocellular carcinoma (44), and non-small cell lung cancer (45). Among the upregulated miRNAs in our results, miR-485-5p has been reported earlier to be associated with atherosclerosis (46), and lung and oral cancer (47, 48), whereas, miR-452-3p has been not reported earlier and might be a novel biomarker. Overall, these differentially expressed miRNAs, significantly correlated with HbA1c in obese patients with metabolic diseases and seem to regulate the glycemic pathways. The mechanisms behind the observed correlations of these miRNAs with HbA1c are still unclear and need to be investigated further.

Another set of miRNAs: miR-106b-3p, miR-652-3p, and miR-93-5p, which were downregulated in OBM subjects, negatively correlated with the creatinine levels in these patients. It has been reported earlier that elevated serum creatinine levels are associated with late stages of diabetic nephropathy or renal damage (49). The role of these miRNAs is either a cause or consequence of renal damage or possible existing hypertension in the OBM cohort. The miR-652-3p has been reported to be relevant for insulin resistance (50) and in polycystic ovary syndrome (PCOS) patients, its expression has been shown to be downregulated in the context of creatinine and HDL and is most likely associated with hepatic involvement in cases of insulin resistance (51). miR-106b-3p has been earlier reported to be downregulated in dengue infection (52); its significance in metabolic disorders is unknown. Given the significant association of miR-106b-3p with both gender (higher expression in females in comparison to males) and smoking status (higher expression in non-smokers compared to smokers) of patients in our dataset, this miRNA needs a more detailed mechanistic investigation as its significant correlation with creatinine might be conditioned on the patient’s sex and smoking status. Our results indicate that miR-652-3p is not only negatively correlated with creatinine but also with high-density lipopolysaccharides (HDL), indicating its possible role in dyslipidemia in obese patients and warrants more investigation. The results from our study indicate decreased expression of miR-130a-3p, miR-130b-5p, miR-363-3p, miR-636, and miR-652-3p respectively in the OBM subjects. Among these miRNAs, miR-363-3p, miR-130b-5p, and miR-636 correlated with cholesterol, and miR-130a-3p correlated with LDL. It has been reported previously that miR-130a-3p levels were elevated in the pancreatic islets of hyperglycemic subjects (53) as well as progressive cardiac failure (54).

In line with previous studies, we report a significant differential expression of miRNAs that play critical roles in insulin resistance, sensitivity, and release. For example, miR-122-5p, miR-221-3p, miR-126-3p, miR-223-3p, and miR-93-5p, which are downregulated in OBM versus OBO, have been described within the context of insulin sensitivity and resistance (55). In addition, miR-34b-3p, miR-9-3p, miR-375, miR-146a-3p, and miR-30e-5p, which are upregulated in OBM versus OBO have been involved in insulin release in pancreatic β-cells and regulate β-cell fate (56–59). Interestingly, IGF1R, a receptor tyrosine kinase that mediates actions of insulin-like growth factor 1 and one of the factors that are altered in obesity is a key target of differentially expressed miRNAs identified by our framework including miR-182-5p. Another important set of targets for the differentially expressed miRNAs were the MAPK genes (MAP3K2 and MAP2K4) which belong to the family of mitogen-activated protein kinase (MAPK). MAPK genes and their interactors have been reported to protect against adverse effects of high-fat feeding in a murine model, demonstrating a decreased weight gain, improved glucose tolerance, and insulin sensitivity, with markedly diminished adipose tissue inflammation (60).

In conclusion, our data show that subjects with morbid obesity and metabolic syndrome compared to individuals with obesity without metabolic syndrome show differential levels of several miRNAs which can regulate multiple genes and metabolic pathways relevant to glycemic regulation, lipid metabolism, and cellular regeneration. However, the cause or consequence merits further studies. The miRNA group associated with metabolic syndrome in morbidly obese subjects may have a pathophysiologic role that warrants further elucidation. Regardless of their role in disease pathogenesis these groups of miRNAs can serve as additional markers to segregate OBM and OBO that can aid divergent management strategies of treatment. To the best of our knowledge, this is the first study of its kind that addresses the role of miRNAs in morbidly obese healthy versus obese metabolic syndrome adults for a population indigenous to Qatar. We do acknowledge that our dataset is small and further studies are warranted in additional larger cohorts to corroborate the importance of the identified differentially expressed miRNAs.

## Data Availability Statement

All the data including processed microRNA and anonymized patient profiles with clinical characteristics as well as the code required to generate the results are available at: https://github.com/raghvendra5688/OBH_vs_OBO_MiRNA_HMC.

## Ethics Statement

The studies involving human participants were reviewed and approved by HMC, IRB protocol #16245/16. The patients/participants provided their written informed consent to participate in this study.

## Author Contributions

FM, RM, FF, and A-BA-S conceived the study. FM, AI, TS, FC, AP, MA, and IA collected, purified, and harmonized the biological samples. RM and EU built the computational pipeline and performed bioinformatics analysis. FF and A-BA-S supervised the analysis. FM, RM, and EU wrote the manuscript. All authors proofread the manuscript. All authors contributed to the article and approved the submitted version.

## Conflict of Interest

Authors FM, AI, TS, AP, MA, IA, and A-BA-S were employed by HMC.

The remaining authors declare that the research was conducted in the absence of any commercial or financial relationships that could be construed as a potential conflict of interest.

## Publisher’s Note

All claims expressed in this article are solely those of the authors and do not necessarily represent those of their affiliated organizations, or those of the publisher, the editors and the reviewers. Any product that may be evaluated in this article, or claim that may be made by its manufacturer, is not guaranteed or endorsed by the publisher.

## Supplementary Material

Supplementary Table 1 Differentially expressed miRNAs with their ΔCR_T_ value, RQ and significance levels (p-value).

Supplementary Table 2 Top five pathways identified to be significantly enriched w.r.t overexpression analysis of mRNAs estimated to be potentially regulated by the differentially expressed miRNAs of interest through the mirDIP portal. The pathways highlighted in bold have relevant literature specifying their role in obesity and metabolic syndrome.

Supplementary Table 3 Top three biological processes (bp), molecular functions (mf) and cellular components (cc) identified to be significantly enriched w.r.t overexpression analysis of mRNAs estimated to be potentially regulated by the differentially expressed miRNAs of interest through the mirDIP portal.
