# Supplementary material for: Characteristic MicroRNAs Linked to Dysregulated Metabolic Pathways in Qatari Adult Subjects With Obesity and Metabolic Syndrome
Source: Front Endocrinol (Lausanne). 2022 Jul 22;13:937089. doi: 10.3389/fendo.2022.937089 (PMC9352892; doi:10.3389/fendo.2022.937089)
Supplement: Supplementary Table 2 — Top five pathways identified to be significantly enriched w.r.t overexpression analysis of mRNAs estimated to be potentially regulated by the differentially expressed miRNAs of interest through the mirDIP portal. The pathways highlighted in bold have relevant literature specifying their role in obesity and metabolic syndrome. [file Table_2.docx]

- MiRNAs are key regulators in metabolic diseases in obese adult patients.
- Metabolically unhealthy obese patients have distinct circulatory microRNA signature.
- 64 miRNAs differentially expressed between obese healthy vs unhealthy patients.
- Differentially expressed miRNAs associate significantly with HBA1c and Creatinine.
- Aberrant MiRNAs regulate multiple genes involved in metabolic pathways.

MiRNAs are key regulators in metabolic diseases in obese adult patients. We identified that metabolically unhealthy obese Qatari patients have distinct circulatory microRNA signature. We determined that 64 miRNAs were differentially expressed between obese healthy vs obese unhealthy patients. Several of these differentially expressed miRNAs associated significantly with clinical traits such as HBA1c (high blood glucose levels) and Creatinine levels. The aberrant miRNAs regulate multiple genes which are involved in metabolic pathways, thereby, providing insight into potential mechanisms of metabolic disorder between obese healthy vs unhealthy obese Qataris.
